# The Capacity to Buffer and Sustain Imbalanced D-Subgenome Chromosomes by the BBAA Component of Hexaploid Wheat Is an Evolved Dominant Trait

**DOI:** 10.3389/fpls.2018.01149

**Published:** 2018-08-07

**Authors:** Xin Deng, Yan Sha, Zhenling Lv, Ying Wu, Ai Zhang, Fang Wang, Bao Liu

**Affiliations:** ^1^Key Laboratory of Molecular Epigenetics of the Ministry of Education (MOE), Northeast Normal University, Changchun, China; ^2^College of Oceanology and Food Science, Quanzhou Normal University, Quanzhou, China

**Keywords:** pentaploidy, aneuploidy, polyploidy, dosage imbalance, buffering capacity, genetic diversity, evolution, *Triticum*

## Abstract

Successful generation of pentaploid wheat (genome, BBAAD) via interspecific hybridization between tetraploid wheat (BBAA) and hexaploid wheat (BBAADD) holds great promise to mutually exchange desirable traits between the two cultivated wheat species, as well as providing a novel facet for evolutionary studies of polyploid wheat. Taking advantage of the viable and fertile nature of an extracted tetraploid wheat (ETW) with a BBAA genome that is virtually identical with the BBAA component of a hexaploid common wheat, and a synthetic hexaploid wheat, we constructed four pentaploid wheats with several distinct yet complementary features, of which harboring homozygous BBAA subgenomes is a common feature. By using a combined FISH/GISH method that enables diagnosing all individual wheat chromosomes, we precisely karyotyped a larger number of cohorts from the immediate progenies of each of the four pentaploid wheats. We found that the BBAA component of hexaploid common wheat possesses a significantly stronger capacity to buffer and sustain imbalanced D genome chromosomes and appears to harbor more structural chromosome variations than the BBAA genome of tetraploid wheat. We also document that this stronger capacity of the hexaploid BBAA subgenomes behaves as a genetically controlled dominant trait. Our findings bear implications to the known greater than expected level of genetic diversity in, and the remarkable adaptability of, hexaploid common wheat as a staple crop of global significance, as well as in using pentaploidy as intermediates for reciprocal introgression of useful traits between tetraploid and hexaploid wheat cultivars.

## Introduction

Aneuploidy, with losses and/or gains of individual chromosomes, and hence deviating from the default balanced chromosome complement(s) of any species, is a large-effect genetic variant with profound biological consequences. Aneuploidy generally causes compromised fitness and is the causation of many important human diseases, e.g., the Down Syndrome due to trisomy of chromosome 21 (Megarbane et al., 2009; Letourneau et al., 2014). However, aneuploidy is not always associated with reduced cellular or organismal fitness. Recent studies have revealed aneuploidy as a major mechanism underlying adaption in unicellular microbes especially under strong selection (Rancati et al., 2008; Pavelka et al., 2010; Kaya et al., 2015; Millet et al., 2015). Also, certain unbalanced karyotypes may promote cellular growth and are principle drivers for the evolution of many types of cancers (Torres et al., 2008; Rutledge and Cimini, 2016; Sansregret and Swanton, 2017). Furthermore, recent studies have documented that aneuploidy is common and often persistent (rather than transit as commonly thought) in newly formed plant polyploids (Xiong et al., 2011; Chester et al., 2012; Zhang et al., 2013), which has led to the suggestion that numerical chromosome changes (aneuploidy) may have played a protracted role at the initial stages of polyploid establishment, adaptation, and evolution (Soltis et al., 2015). This possibility is bolstered by the observation that many types of aneuploidy are reversible in the sense that euploidy progenitors can be readily generated from aneuploid progenitors, which however may retain some of the desirable properties of the aneuploid progenitors (Henry et al., 2010). This is consistent with our recent observation that aneuploidy-induced epigenetic modifications in the form of altered DNA methylation were imparted to the aneuploidy-derived euploid progenies at appreciable frequencies (Gao et al., 2016).

The capacity to harbor additional chromosome(s) in the form of aneuploidy is a property intrinsically different between species. For example, plants in general are more tolerant to an imbalanced genome composition than animals (Matzke et al., 2003; Henry et al., 2005). Within a given species, a polyploid genome is more permissive to unbalanced chromosomes (especially chromosome loss) than its diploid or haploid counterparts (Ramsey and Schemske, 1998; Birchler et al., 2001; Wu et al., 2018). Indeed, in several polyploid crops, such as common wheat, complete sets of aneuploidies can be generated and maintained (Sears, 1944).

A recent study documented that there exists a fundamental difference between laboratory strains and wild collections in budding yeast (*Saccharomyces cerevisiae*) with respect to their capacities to harbor additional chromosomes, i.e., being aneuploidy (Hose et al., 2015). Specifically, it was found that laboratory strains of *S. cerevisiae* were poorly tolerant to numerical chromosome variation (NCV), while their wild counterparts showed little detrimental impacts when extra chromosome were present (Hose et al., 2015). A major mechanism underlying this disparity in harboring an additional chromosome between the laboratory and wild yeast strains is due to their difference in dosage compensation whereby expression level of genes encoded by the extra chromosome can be attenuated to that of the euploidy in wild but not in laboratory strains (Gasch et al., 2016; but see Torres et al., 2016). This finding in yeast is reminiscent of earlier studies in *Arabidopsis thaliana*, which already documented that a locus named *SDI* (sensitive to dosage imbalance) located on chromosome 1 affects ploidy-dependent transmission distortion, has a role in aneuploid viability, and hence impacts chromosome composition of triploid-derived progeny cohorts (Henry et al., 2005, 2007). Together, it is clear that the capacity to buffer and retain extra chromosome(s), i.e., differences in sustaining the severity and diversity of aneuploidies, is a genetically controlled trait in a given organism, and the phenotypic penetrance of which is likely ploidy level-dependent. However, the issue remains understudied in any organism.

Hexaploid common wheat (*Triticum aestivum* L., genome BBAADD) is a very young (*ca.* 8,500 year-old) species formed by allopolyploidization [hybridization and whole genome duplication (WGD)] between tetraploid emmer wheat (*Triticum turgidum*, genome BBAA) and diploid *Aegilops tauschii* (genome DD) (Kihara, 1944; McFadden and Sears, 1946; Feldman et al., 1995; Huang et al., 2002). Nearly a century ago, the pioneering works by Sax and Kihara have independently demonstrated that hexaploid common wheat and tetraploid emmer wheat could be hybridized to produce fertile pentaploid hybrids (genome BBAAD) (Sax, 1922; Kihara, 1925). Recent years have witnessed a renewed interest in the generation of pentaploid wheat for the purpose of reciprocally introgressing desirable traits from one species to the other (reviewed in Padmanaban et al., 2017b, 2018). Apart from the practical success (Wang et al., 2005; Eberhard et al., 2010; Padmanaban et al., 2017a,b), an interesting observation is that, in the progenies of pentaploid wheat, there exists a significant positive correlation between proportions of the hexaploid wheat BBAA genomic content and the retention of unbalanced D chromosome (Martin et al., 2011; Padmanaban et al., 2017a). This finding suggests that the BBAA components of hexaploid wheat have a stronger capacity to retain unbalanced D chromosomes than that of the tetraploid wheat BBAA genome. However, the experimental design in these studies, being breeding-oriented, all involved heterozygous BBAA genomes due to combining different genotypes of hexaploid and tetraploid wheat, and hence, does not allow a direct comparison to reach a confirmative conclusion.

With largely intact subgenomes, as well as having both progenitor species still being in extant, it is possible to extract the BBAA component from a given hexaploid wheat cultivar, by hybridization with a *durum* wheat and repeated backcrossing with the hexaploid wheat as a recurrent parent, to reconstitute an “extracted” tetraploid wheat (Kerber, 1964). The extracted tetraploid wheat (ETW) is viable and partially fertile although with severe pleiotropic growth and development abnormalities (Kerber, 1964; Zhang et al., 2014; Liu et al., 2015). Thus, crossing a hexaploid common wheat genotype, from which the ETW was extracted, with ETW will generate a pentaploid wheat with homozygous BBAA subgenomes representing that of the hexaploid wheat. Accordingly, crossing a synthetic hexaploid with the same tetraploid wheat cultivar whereby the hexaploid wheat was produced will generate pentaploid wheat with homozygous BBAA genomes representing that of the tetraploid wheat genome. The present study was designed according to this rational, along with additional considerations, to construct four pentaploid wheat lines with distinct features that are suitable to specifically address the question, i.e., whether the capacity to buffer and sustain imbalanced D-genome chromosomes by the BBAA component of hexaploid wheat is an evolved trait. Our results, based on high resolution FISH/GISH karyotyping of large numbers of immediate progeny cohorts of each of the four pentaploid wheat lines, have confirmed the previously only implied possibility (Martin et al., 2011; Padmanaban et al., 2017a). Our results also provide additional insights into the extent and trend of numerical and structural chromosome instabilities in the pentaploid wheat-derived progenies.

## Materials and Methods

### Plant Materials

We used two genotypes of tetraploid wheat and three genotypes of hexaploid wheat to construct four pentaploid wheat lines (genome BBAAD, 2*n* = 35). TTR13 (*T. turgidum*, ssp. *durum*, BBAA, 2*n* = 28) represents the natural *durum* wheat. The ETW (BBAA, 2*n* = 28) represents the BBAA component of a natural hexaploid bread wheat (*T. aestivum* L., BBAADD, 2*n* = 42) cultivar, TAA10, as detailed previously (Kerber, 1964; Zhang et al., 2014). By hybridization with a *durum* wheat, and then recurrent backcrossing with TAA10 for 9-times (Zhang et al., 2014), ETW could be regarded as BBAA component of TAA10, as they are >98% identical based on recombination and Mendelian inheritance (Zhang et al., 2014). XX329 (2*n* = 42, BBAADD) is a resynthesized hexaploid wheat line by hybridizing and doubling hybrid of ETW and TQ18 (*A. tauschii*, 2*n* = 14, DD) (Zhang et al., 2014). Synthetic hexaploid wheat (SHW) (BBAADD, 2*n* = 42) represents a newly synthesized hexaploid wheat lines by crossing TTR13 with TQ18 followed by spontaneous genome doubling of the F1 hybrid.

Four independent pentaploid wheat lines were separately generated from the hexaploid × tetraploid combinations. These include pentaploid XE (XX329 × ETW), pentaploid TE (TAA10 × ETW), pentaploid TT (TAA10 × TTR13) and pentaploid ST (SHW × TTR13), and in all four lines, the hexaploid wheat was used as the maternal parent. All pentaploid wheat individuals were obtained by embryo rescue by inoculating the inter-ploidy F1 hybrid immature embryos on Murashige and Skoog (MS) medium (PhytoTechnology, M519) at a constant temperature of 25°C. The germinated seedlings were transplanted to soil when they were 6–7 cm in height.

Seeds were harvested from several individuals of each pentaploid wheat line. All grown seedlings from the germinated seeds of each progeny individual of the pentaploid wheats were referred to as immediate selfed progenies. They were transferred to pots containing nutrient-sufficient soil under normal greenhouse conditions of constant 25°C with 16/8 h light/dark photoperiod. Root-tips were sampled for cytological analysis, while the plants were grown to maturity for phenotyping.

### Chromosome Preparation and Fluorescence *in Situ* Hybridization

Roots about 1.5∼2.0 cm long were taken from the seedlings and treated in N_2_O gas for 2 h. These roots were then fixed in 90% (v/v) acetic acid and stored in 75% (v/v) alcohol at -20°C until use. Mitotic spread chromosome slides were prepared from the root-tips according to Zhang et al. (2013). For each plant, chromosomes were counted for at least five well-spread metaphase cells.

Sequential florescence *in situ* hybridization (FISH) and genomic *in situ* hybridization (GISH) were performed according to a protocol reported previously (Zhang et al., 2013) with minor modifications. Briefly, Texas red-5-dCTP (PerkinElmer, NEL426001EA) was used for labeling the repeated sequence clone pAs1 (Rayburn and Gill, 1986) and genomic DNA isolated from *A. tauschii*, respectively. ChromaTide^TM^ Alexa Fluor^TM^ 488-5-dUTP (Thermo Fisher, C11397) was used for labeling the rye repeated sequence clone pSc119.2 and genomic DNA from *T. urartu*, respectively. In GISH, genomic DNA of *A. bicornis* was used as blocker. In both FISH and GISH, DAPI (Vector, H-1200) was also used to counterstain the chromosomes.

Both the FISH and GISH slides were examined under an Olympus BX63 fluorescence microscope and captured by Q-capture imaging software (QImaging, Version 2.90.1). Brightness, contrast and background were adjusted as an entirety in Adobe Photoshop CC.

### Phenotyping

Nine morphological traits, including plant height, tiller number, stem diameter, flag-leaf width, spike length, spike density, seed length, seed width, and seed set, were phenotyped for at least three individuals from each of the pentaploid wheat lines. Plant height, tiller number, stem diameter, and flag-leaf width were measured at the mature stage. Stem diameter was represented by the maximum value of stems at 1.5 cm below the last node. Spike length, seed length, and seed width were measured after harvest. The longest spike was used to measure the spike length and spikelet number. Spikelet density was calculated through spikelet number divided by the corresponding spike length. We also used the maximum value of seed set per spike to represent seed set of a given plant. Seed set was the total seed number of two base florets relative to the total number of two base florets. Seed length and width were represented by the average values of the same 10 seeds.

### Data Analysis and Statistical Test

Statistical test of each of the data comparisons was performed in R software (version 3.4.0). Vioplots of chromosome number distribution was depicted using R packages of vioplot (Hintze and Nelson, 1998). Karyotypes of each plant were depicted by R packages of pheatmap^1^. The Student’s *t*-test and F-test were used to interrogate whether the capacities to buffer and sustain unbalanced D chromosomes by the BBAA component were significantly different among the four pentaploid wheat lines. A prop.test was performed to determine possible differences in composition of the D chromosomes within a given pentaploid wheat line via pairwise comparisons between any two D chromosomes. A Wilcoxon test was used to determine differences in each of the nine morphological traits among the four pentaploid wheat lines.

## Results

### Features of the Four Constructed Pentaploid Wheat Lines

**FIGURE 1:**
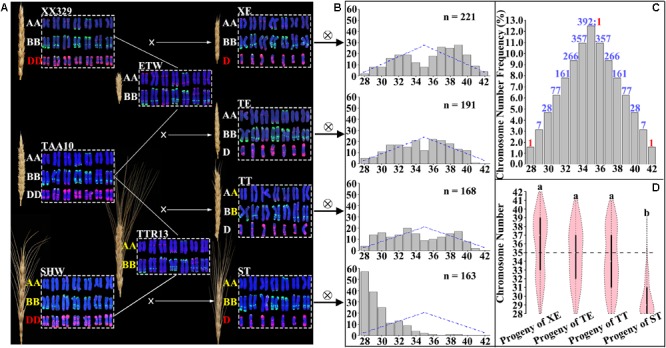
Four different pentaploid wheat lines generated by crossing three hexaploid wheat genotypes with two tetraploid wheat genotypes, and the overall chromosome number distributions in the immediate progeny swarms of the pentaploids. **(A)** FISH-based karyotypes and spike morphology of hexaploid wheat, tetraploid wheat and pentaploid wheat. PSc119.2 (green coloration) and pAs1 (red coloration) were used as FISH probes. Subgenome designations **(A,B,D)** in the same and different colored fonts denote that they were from the same source or difference sources. **(B)** Theoretically expected (blue lines) and observed (gray bar) plant numbers in each of the immediate progenies of the four pentaploid wheat lines. Expected values were obtained by transforming the total karyotyped plant number according to the expected frequency in **(C)**. The numbers of observed (karyotyped) individuals in each F2 population are given. The x-axis is the chromosome number. **(C)** Theoretical (expected) chromosome number distribution in the immediate progeny swarms of pentaploid wheat. The numbers on the top of the gray bars refer to the expected values in each of the chromosome number categories belonging to either aneuploidy (colored blue) or euploidy (colored red). **(D)** Comparison of the overall chromosome number distributions among the immediate progeny swarms of the four pentaploid wheat lines. Letters above each vioplots denote statistical significance of pairwise comparisons (Var. test, *P*-values < 0.05).

We produced four lines of pentaploid wheat (genome BBAAD) using three hexaploid wheat genotypes (genome BBAADD) and two tetraploid wheat genotypes (genome BBAA) (**Figure 1A**). As such, three pentaploid wheat lines (designated XE, TE, and ST) harbor *homozygous* BBAA genomes plus a single D genome (**Figure 1A**). This is because the maternal hexaploid wheat genotype and paternal tetraploid genotype for each of these three pentaploid lines contained the same BBAA component (**Figure 1A**). The fourth pentaploid line (designated TT) harbors heterozygous BBAA genome with the BBAA components being a F1 hybrid between the maternal hexaploid genotype and the paternal tetraploid genotype (**Figure 1A**). Two additional features characterize the three pentaploid wheat lines with homozygous BBAA genomes. First, the D genome in two pentaploid lines, XE and TE, are from different *A. tauschii* accessions; second, pentaploid lines XE and ST share the same D genome while their homozygous BBAA genomes are different (**Figure 1A**). Together, characteristics of these four purposely constructed pentaploid lines render them suitable for addressing the primary objective of this study, i.e., weather the capacity to buffer and sustain imbalanced D-genome chromosomes by the BBAA component of hexaploid wheat is an evolved trait.

### Similarity and Difference in Overall Chromosome Number Distribution by the Immediate Selfed Progenies of the Four Pentaploid Wheat Lines

As described above, we selfed each of the four pentaploid lines (XE, TE, TT, and ST) (**Figure 1A**) to produce their immediate progeny swarms (**Figure 1B**). We karyotyped 234, 196, 181, and 169 progeny individuals of XE, TE, TT, and ST, respectively, by the sequential FISH/GISH karyotyping protocol, which enables reliable diagnosis of each of the wheat chromosomes (Zhang et al., 2013). The overall chromosome number distribution in the four pentaploid progeny swarms, each as a population, was tabulated. Data showed that the chromosome distributions in progeny swarms from three (XE, TE, and TT) of the four pentaploid lines are similar, but that of the fourth pentaploids (ST) is strikingly different (**Figure 1B**). Specifically, while chromosome numbers in the progeny swarms of XE, TE, and TT showed more or less similar quantities of individuals with chromosome numbers fewer or more than 2*n* = 35, those of ST substantially biased toward individuals (93.5%) with chromosome numbers fewer than 2*n* = 35, moreover, 33.7% of the plants have reverted to tetraploid wheat, i.e., with all D chromosomes being eliminated (**Figure 1B**). Expectedly, in the progeny swarms of all four pentaploid lines, chromosomes of the A and B subgenomes were found to be stable (i.e., they all contained seven pairs of chromosomes for each subgenome) in great majority (>92.8%) of the plants; the small proportions of plants with one or more BBAA chromosomes being in an aneuploid state and/or with structural variations in each population are described separately in later sections and not included here. Thus, only the D subgenome chromosomes are variable and contributing to differences in the overall chromosome number distribution. Taken together, these observations suggest that while the BBAA components of three pentaploids, XE, TE, and TT, showed similar and strong capacities to buffer and sustain unbalanced D chromosomes, this capacity by the BBAA component of ST is significantly weaker (*T*-test, both *P*-values < 0.05 in the comparisons of XE versus ST, TE versus ST, and TT versus ST).

In theory, assuming random assortment of the unpaired D chromosomes during formation of male and female gametes in the pentaploid lines, the chromosome numbers of their progeny swarms should range from 28 (reverting to tetraploid wheat) to 42 (converting to hexaploid wheat) (**Figure 1C**). Compared to this theoretical chromosome number distribution, the observed chromosome numbers in the progeny swarms of three pentaploid lines (XE, TT, and ST) all showed significant deviation (*χ*^2^ = 50.94, d.f. = 14, *P*-value = 4.23e-06 for progenies of XE; *χ*^2^ = 46.22, d.f. = 14, *P*-value = 2.58e-05 for progenies of TT; *χ*^2^ = 1524.90, d.f. = 14, *P*-value < 2.2e-16 for progenies of ST). Interestingly, chromosome number distribution in progeny swarms of the pentaploid line TE was not significantly deviating from the theoretically expected distribution (*χ*^2^ = 20.13, d.f. = 14, *P*-value = 0.13), suggesting a strong parental combination difference. In any case, although the three pentaploid lines (XE, TE, and TT) are different with respect to their conformities to the theoretical chromosome number distribution in their progeny swarms, their BBAA components manifested similar capacities to buffer and sustain the unbalanced D chromosomes, and which was significantly different from that of ST (**Figure 1D**).

### Similarity and Difference in Chromosome Composition of the Immediate Selfed Progenies of the Four Pentaploid Wheat Lines

**FIGURE 2:**
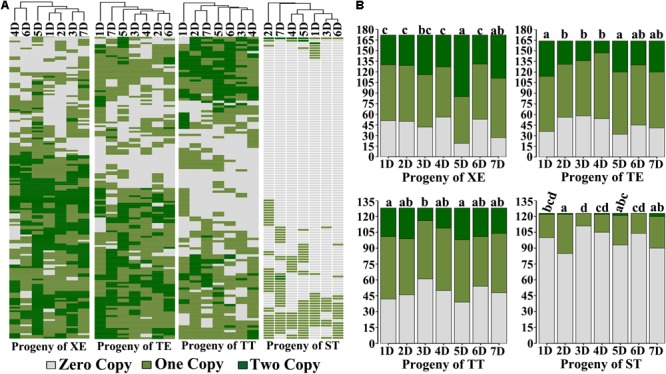
Exact D-subgenome chromosome composition in the immediate progeny swarms of the four pentaploid wheat lines. **(A)** Heatmaps illustrating the D chromosome composition in each karyotyped cohort of the immediate selfed progeny swarms of the four pentaploid wheat lines. **(B)** Bar-plots showing the summarized copy number for each of the seven D chromosomes (1D–7D) in the immediate selfed progeny swarms of the four pentaploid wheat lines. The small letter(s) above each bar plot denotes for statistical difference of pairwise comparisons (Prop.test, *P*-values < 0.05). The y-axis in both **(A,B)** represents the number of karyotyped cohorts, while the x-axis is the seven D chromosomes.

The foregoing results concern the overall chromosome number distributions in progeny swarms of the four pentaploid lines. We further analyzed the exact chromosome compositions in each of these progeny plants. Because all plants for this analysis contained the complete A- and B-subgenome chromosomes, described above, the only variable is the number of D-subgenome chromosomes. Data showed that almost each D chromosome-containing progeny individual of all four pentaploid lines contained a different “D chromosome composition” configured by both individuality, and copy number thereof, of the harbored D chromosome(s) (**Figure 2A**). Consequently, vast karyotypic diversity was seen in these progeny plants due to NCV of the D chromosome(s), with those of pentaploids XE, TE, and TT being markedly more diverse than those of ST (**Figure 2A**), which is consistent with the distributions of overall chromosome numbers (**Figure 1D**). An additional feature characteristic of the progeny plants of pentaploids XE, TE, and TT is that their substantial proportions contained two copies of the D chromosomes while this situation was rare in progeny plants of ST (**Figure 2A**).

We found that occurrence of the D-subgenome chromosomes was not equal in the progeny plants of a given pentaploid line (**Figure 2B**). For progeny plants of XE, chromosome 5D was significantly overrepresented relative to the rest five D chromosomes, i.e., 1D, 2D, 3D, 4D, and 6D, among which the difference was not statistically significant; for progeny plants of TE, chromosomes 1D and 5D were significantly overrepresented than chromosomes 2D, 3D, and 4D, while no difference was detected in pairwise comparisons concerning the rest D chromosomes; for progeny plants of TT, chromosomes 1D and 5D were significantly overrepresented than chromosome 3D, while no difference was detected in the rest pairwise comparisons; for progeny plants of ST, chromosomes 2D was significantly overrepresented than chromosomes 1D, 3D, 4D, and 6D, while no difference was detected in the pairwise comparisons for the rest D chromosomes. However, all D chromosomes occurred at low frequencies in the progeny plants of ST relative to those of the other three pentaploid lines (**Figure 2B**). Notably, for still unknown reasons, chromosome 5D was significantly overrepresented in progenies of the three pentaploid wheats (XE, TE, and TT). Another striking feature is, in the progeny plants of all four pentaploid lines, the 1 copy versus 2 copies for a given retained D chromosome appeared proportional (**Figure 2B**), suggesting that if selection has been in action at the gametophytic stage, then it has been unbiased between the female and male gametes, an issue warrants further investigations.

### Difference in the Capacity to Buffer and Sustain Imbalanced D-Genome Chromosomes by the BBAA Components of Hexaploid Wheat With Different Evolutionary Histories

The above results have shown that both the overall chromosome number distributions and chromosome compositions are similar among the immediate progeny swarms of the three pentaploids, XE, TE, and TT, but which are strikingly different from those of ST (**Figures 1A,B**). Naturally, we analyzed these differences in relation to the BBAA components constituting the pentaploid lines, which have different evolutionary histories. Specifically, of the three pentaploid wheat lines (XE, TE, and ST) harboring homozygous BBAA genomes (**Figure 1A**), two (XE and TE) contained the BBAA component of a common wheat, cv. TAA10; this component was extracted from TAA10 by hybridization and repeated backcrossing with TAA10 (Kerber, 1964; Zhang et al., 2014), and hence was termed ETW (Zhang et al., 2014). Because the hexaploid wheat XX329 is a resynthesized line via allohexaploidization (hybridization coupled with WGD) between ETW and *A. tauschii* (accession TQ18) (Zhang et al., 2014), the D genome in the derived pentaploid wheat XE is of diploid *A. tauschii* origin (accession TQ18). In contrast, the D genome in TE was that of the original common wheat TAA10 (**Figure 1A**). The third pentaploid line harboring homozygous BBAA genomes is ST (**Figure 1A**). The hexaploid parent SHW (synthetic hexaploid wheat) was a synthetic line constructed via allohexaploidization between *durum* wheat cv. TTR13 and *A. tauschii* (accession TQ18), hence the BBAA component in ST was the same as TTR13 while the D genome was that of TQ18, i.e., the same as that in XE (**Figure 1A**). Thus, while the BBAA components of pentaploid lines XE and TE are of domesticated common wheat (cv. TAA10), that of ST is the tetraploid *durum* wheat (cv. TTR13). A fundamental difference between the BBAA component of hexaploid common wheat and the BBAA genomes of *durum* wheat is that the former has been co-evolved with the D subgenome for *ca.* 8,500 years since speciation of common wheat, *T. aestivum* L. (Kihara, 1944; McFadden and Sears, 1946; Feldman et al., 1995; Huang et al., 2002), while the later has never been co-exiting with the D genome. Therefore, our observation that the BBAA component in pentaploid lines XE and TE has a strong capacity to buffer and sustain unbalanced D chromosomes, while that in pentaploid line ST has a much weaker capacity can be most parsimoniously explained by their different properties, i.e., the former has co-evolved with the D subgenome, while the later has not. The BBAA component of pentaploid TT was a F1 hybrid between that of TAA10 and TTR13. The F1 hybrid BBAA component of TT showed a similar strong capacity to buffer and sustains the unbalanced D chromosomes as shown by that of XE and TE. This suggests that the capacity to buffer and sustain imbalanced D-chromosomes by the BBAA components of hexaploid wheat is a dominant trait.

The above said, an alternative possibility concerns whether nature of the D genome chromosomes *per se* may also play a role in determining their retention versus elimination. Intuitively, like the BBAA genomes, the presumably co-evolved D chromosomes may also have adapted to become more compatible with the BBAA subgenome that those of *A. tauschii* that have never been coexisting in the same nucleus/cytoplasm with the BBAA genomes. As described above, the fact that there was no overt difference between XE that contained the D genome of *A. tauschii* (TQ18) from the two pentaploid lines (TE and TT) that harbored the co-evolved D genome with respect to the particular phenotypic manifestation (the capacity to buffer and sustain imbalanced D-chromosomes) clearly ruled out this possibility, and hence further reaffirms our scenario that the evolved dominant trait was encoded by the BBAA genomes.

### Numerical Chromosome Variations Also Occurred in the A- and B-Subgenome Chromosomes in Progenies of the Pentaploid Wheat Lines

We also detected NCVs, i.e., aneuploidy, concerning the A and B chromosomes in small proportions of the progeny swarms of all four pentaploid wheat lines (Supplementary Table S1). The NCVs may involve gain or loss of one or more chromosomes of either the A or B subgenome, or simultaneous gain and loss of chromosomes involving both A and B subgenomes (Supplementary Table S1). Notably, sometimes, aneuploidies of the A and/or B chromosomes were accompanied with gain of extra D chromosomes (i.e., one D chromosomes being at three copies). Collectively, the A and B chromosome aneuploidies accounted for 5.1% (12 of 234), 2.0% (4 of 196), 7.2% (13 of 181), and 3.0% (5 of 169) of the karyotyped progeny plants derived from the XE, TE, TT, and ST pentaploid lines (Supplementary Table S1).

### Structural Chromosome Variations Involving All Three Subgenomes Occurred in Progenies of the Pentaploid Wheat Lines

**FIGURE 3:**
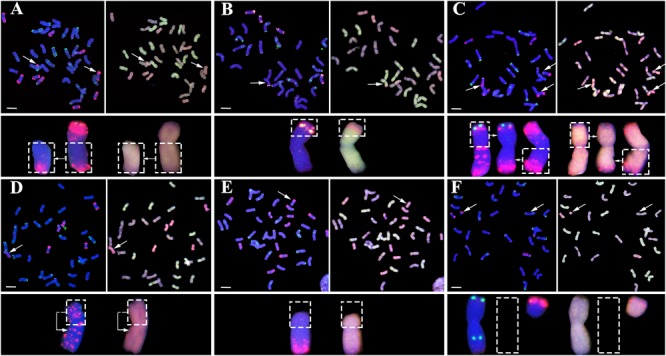
Representative structural chromosome variations (SCVs) in the immediate progenies of the pentaploid wheat lines. **(A)** Telocentrics, **(B)** Inter-subgenome translocations, **(C)** Intra-subgenome translocations, **(D)** Isochromosomes, **(E)** Truncations, and **(F)** Telocentrics coupled with monosomic 2B (loss of one 2B chromosome). In **(A)** through **(F)**, the FISH images (left of each panel pair) are signals of pSc119.2 (green) and pAs1 (red), while the GISH images (right of each panel pair) are the signals of A subgenome (green), D subgenome (red), and B subgenome (blue), respectively. Structural variant chromosomes (marked by arrows) are zoomed in and shown below the images accordingly; while the dashed-lined empty frames in **(F)** refer to loss of one 2B chromosome (monosomic 2B). In all images, the bars = 10 μm.

**FIGURE 4:**
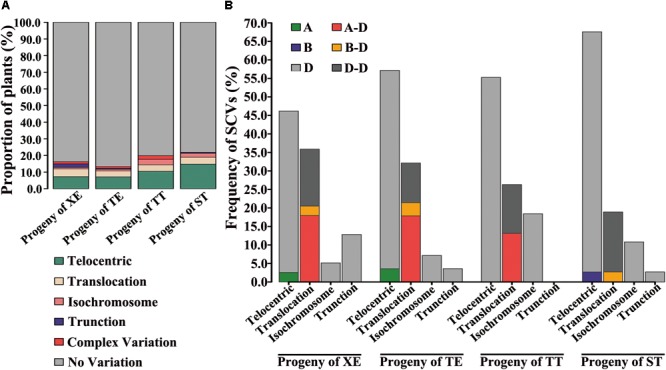
SCVs in the immediate progeny swarms of each of the four pentaploid wheat lines. **(A)** Relative proportions of plants with the variable types of SCVs in the four immediate progeny swarms of the pentaploid wheat lines. **(B)** Relative proportions of chromosomes of each subgenome with SCVs in the four immediate progeny swarms of the pentaploid wheat lines.

Variable types of structural chromosome variations (SCVs) were detected in certain proportions of the karyotyped progeny individuals from all four pentaploid lines. These were found to include telocentrics, translocations, isochromosomes, truncations, as well as complex structural variations containing more than one type of SCVs involving multiple chromosomes (**Figure 3**). Collectively, 16.2% (38 of 234), 13.3% (26 of 196), 19.9% (36 of 181), and 21.9% (37 of 169) of the karyotyped progeny plants from the XE, TE, TT, and ST pentaploid lines, respectively, were found to contain one or more SCVs (**Figure 4A**). Telocentrics were the most abundant type of SCVs, followed by translocations (**Figure 4A**); the two type of SCVs accounted for greater than 72.22% (26 of 36 in TT) and were significantly more abundant than the other SCV types in all four pentaploid wheat progeny populations (Prop.test, all *P*-values < 0.05).

Because more than one SCV may occur in a given individual, we further tabulated numbers of the various types of SCVs in the progeny swarms of each of the four pentaploid wheat lines. In each SCV types, we divided the number of SCVs belonging to intra or inter subgenome by the total number of SCVs to get the frequency of SCVs. We found that great majority of telocentrics involved the D chromosomes in progenies of all four pentaploid lines (**Figure 4B**), as expected given the propensity of unpaired univalents to undergo mis-division at anaphase of meiosis II. Actually, except for 4DL, telocentrics were found for both the long- and short-arm of all seven D chromosomes concerning all four pentaploid lines (Supplementary Table S2). Also as expected, isochromosomes were detected for many of the D chromosome arms, including 2DS, 2DL, 3DS, 3DL, 5DL, 6DS, and 7DS (e.g., **Figure 4B** and Supplementary Table S2). A less expected type of SCVs concerning the D chromosomes was intra-subgenome translocations, which were detected between chromosomes 1D and 2D, 1D and 3D, 1D and 4D, 1D and 7D, 2D and 3D, 2D and 4D, 2D and 6D, 2D and 7D, 4D and 7D, 5D and 6D, and 6D and 7D (e.g., **Figure 3A** and Supplementary Table S2).

Structural chromosome variation were not confined to the D chromosomes. For example, telocentrics of chromosomes 1AS, 4AS, and 5BS were also observed in progeny individuals of TE, XE, and ST, respectively. Moreover, inter-subgenome translocations involving A-D and/or B-D chromosomes were also observed in progeny plants of all the four pentaploid wheat lines. A striking feature of SCVs associated with progenies of ST relative to the other three pentaploid lines was that most of the translocations (six out of seven) were between chromosomes within the D subgenome (**Figure 4B** and Supplementary Table S2). By contrast, majority of the translocations in the other three pentaploid lines (XE, TE, and TT) was inter-subgenomic (**Figure 4B** and Supplementary Table S2).

### Phenotypes of the Pentaploid Wheat Lines

**FIGURE 5:**
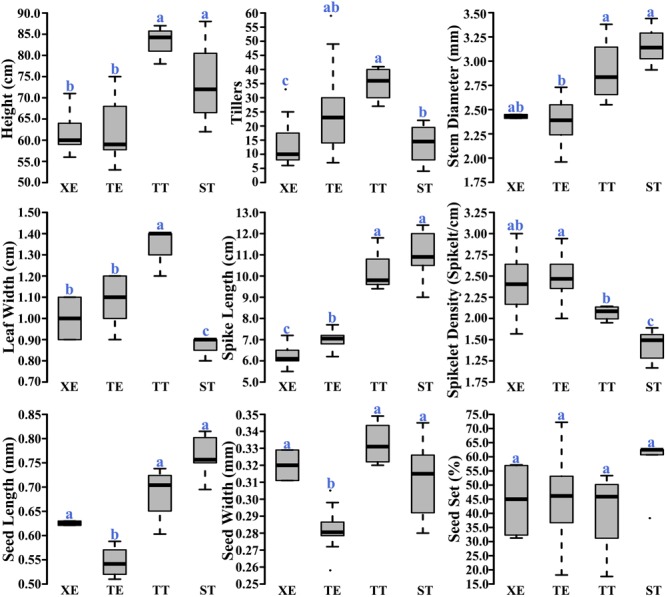
Comparisons of the nine phenotypic traits among the four immediate progeny swarms of the pentaploid wheat lines. Letters above the boxplots denote statistical significance of the pairwise comparisons (Wilcox. test, *P*-value < 0.05).

We measured nine phenotypic traits for each of the four pentaploid wheat lines. We found significant differences for some, but not all, of the phenotyped traits among the lines (**Figure 5**). Also, the phenotypic differences were not consistent across the four lines, that is, a given line was not inferior to others in all traits (**Figure 5**). In particular, we did not find statistical differences among the four lines in seed-setting (**Figure 5**), a major reflection of reproductive fitness. Together, the phenotypic data suggest that the different capacities to buffer and sustain the unbalanced D chromosomes by the BBAA components of the pentaploid wheat lines are probably not related to the performance (fitness) of the pentaploids themselves.

## Discussion

We have shown in this study that all three manifestations, i.e., overall chromosome number distribution/exact chromosome composition (concerning the D subgenome chromosomes), NCVs (concerning the A and B subgenomes), and SCVs (concerning all three subgenomes), are strikingly different between the immediate progenies of a pentaploid wheat (ST) with the BBAA genome of a tetraploid *durum* wheat (cv. TTR13) and two pentaploid wheats (XE and TE) with the BBAA component of a hexaploid common wheat (cv. TAA10). Given the common feature of these three pentaploid wheat lines, i.e., all harboring *homozygous* BBAA subgenomes (**Figure 1A**), our results have unequivocally shown that the BBAA component of a hexaploid wheat possess a greater capacity than the BBAA genome of a *durum* wheat to buffer and sustain unbalanced D chromosomes, and hence, to constitute more kinds, more complex, greater severity of aneuploidies, as well as higher levels of SCVs in the progeny cohorts of the corresponding pentaploid wheats constituted by the former than that by the later. The observation that the immediate progenies of the pentaploid wheat (TT) with its BBAA subgenomes as a F1 hybrid between that of the hexaploid wheat (TAA10) and the tetraploid *durum* wheat (TTR13) does not differ from the two pentaploid wheats (XE and TE) with homozygous BBAA component of the hexaploid wheat indicates that the trait (capacity to buffer and sustain unbalanced D chromosomes) is genetically dominant. The gene(s) responsible for this trait has to be encoded by the A and/or B subgenomes, because no discernible difference was observed with respect to the three manifestations (above) between pentaploid wheats XE and TE which possess the same BBAA subgenomes (both are the BBAA component of hexaploid wheat TAA10) but with different sources of the D genomes, one being the original of TAA10 while the other being of *A. tauschii*, accession TQ18 (**Figure 1A**). Our results are consistent with an earlier observation that in segregating progenies of given pentaploid wheat, those individuals with more BBAA alleles from the hexaploid parent were trended to contain more D chromosomes than those with more alleles from the *durum* wheat (Martin et al., 2011; Padmanaban et al., 2017a, 2018). However, because the pentaploid wheats constructed by these prior studies, being breeding oriented, are all with *heterozygous* BBAA genomes (hybrids between different genotypes of hexaploid wheat and *durum* wheat), other confounding factors, e.g., heterosis in the concerned trait, cannot be ruled out, and hence, an affirmative conclusion cannot be reached. Therefore, our empirical results have confirmed the previously only implicated possibility. Our results are also reminiscent of the earlier studies concerning chromosome compositions of selfed progenies derived from *Arabidopsis thaliana* intraspecific triploids (genome designations are CCC and CWW, respectively) constructed by crossing the same diploid line (Col-0, genome CC) with two different tetraploids, a natural ecotype (Warschau-1, genome WWWW), and an induced line (4x-Col, genome CCCC); it was found that progenies of the CWW triploid showed substantially more complex and extreme aneuploidies than those of CCC (Henry et al., 2005, 2007). However, there is a distinct difference between the *A. thaliana* triploids and the wheat pentaploids, because genomes in the former are from difference ecotypes (i.e., intraspecific) rather than different species, and therefore they are autotriploids.

What might be the mechanistic basis underlying this dominant trait evolved in the BBAA subgenomes of hexaploid common wheat? As proposed in the *A. thaliana* study (Henry et al., 2007), it has to do with buffering against dosage imbalance of genes and their products. Conceivably, this can be accomplished by either or both of dosage compensation and dosage insensitivity. In case of wheat, it is intuitive, as well as experimentally documented, that the BBAA component of hexaploid common wheat possesses an improved (enhanced) version for either or both of these mechanisms. First, given its evolutionary history that the BBAA component has been coexisting and presumably co-evolving with the D subgenome for *ca.* 8,500 years (Kihara, 1944; McFadden and Sears, 1946; Feldman et al., 1995; Huang et al., 2002), naturally, the three subgenomes have been compatible with each other, and therefore incompatibility at each level (structural, epigenetic, or transcriptomic) has been resolved, for example, by rapid genetic and epigenetic changes and rewired gene expression (Kashkush et al., 2002, 2003; Zhao et al., 2011; Feldman and Levy, 2012; Zhang et al., 2016). Second, being at a higher ploidy level, a stronger compensatory capacity at least for WGD has conceivably been reinforced. Third, a strong compensation for dosage imbalance was found to exist in hexaploid common wheat, at least at the RNA transcript level, evidenced in various types of whole-chromosome aneuploidies (Zhang et al., 2017). Forth, it was found that hexaploid wheat formation (synthetics) at the initial stages is not different from neopolyploids of other studied plant species (e.g., Xiong et al., 2011; Chester et al., 2012) in that it is also associated with persistent whole chromosome aneuploidies (Zhang et al., 2013). However, it should be cautioned that properties of the original founder stand(s) of a primitively domesticated form of the tetraploid emmer wheat, *T. turgidum*, which served as the tetraploid maternal parent to give rise to the hexaploid wheat (*T. aestivum*) may differ from the current tetraploid wheats used to reconstruct the synthetic hexaploid wheats (Li et al., 2015). Nevertheless, assuming they have shared the same property, i.e., being associated with persistent aneuploidies, then it is conceivable that the BBAA component of hexaploid common wheat would have not only experienced WGD but also aneuploidy, therefore naturally, it has evolved the capacity to better buffer for not only the potential negative effects of balanced dosage change (due to WGD) but also imbalanced dosage (aneuploidy). Taken together, it is convincing that the BBAA component of hexaploid common wheat has evolved a stronger capacity to buffer and sustain imbalanced D chromosomes and SCVs than the BBAA genome of tetraploid wheat, as shown in this study.

What evolutionary advantages might have been bestowed to hexaploid common wheat by evolving a stronger capacity to buffer and sustain imbalanced D chromosomes and be more inclusive to numerical and SCVs in general? We consider this trait indeed matters. An intrinsic problem associated with polyploid speciation is genetic bottleneck, because very limited progenitor founder stands (if not only one) should have been involved in the initial polyploidization event under most natural settings. Thus, it is surprising that common wheat (*T. aestivum*) as a very young allohexaploid species was found to harbor a level of genetic diversity that is much greater than expected (Dubcovsky and Dvorak, 2007). Among others, one plausible scenario proposed is that both within the geographic region of its origin, known as the Fertile Crescent in the Near East (Salamini et al., 2002), and during its human-mediated global dispersal, hybridizations with wild and/or domesticated tetraploid emmer wheat (*T. turgidum*) might have been frequent when the latter was within pollinating adjacency (Dubcovsky and Dvorak, 2007). For these inter-ploidal hybridizations to occur and being evolutionarily consequential, fitness, fecundity as well as karyotype heterogeneity (chromosome composition) of the successive progenies descended from the pentaploid intermediates would have been critical to enable an eventual return to euploid hexaploidy. Expectedly, a stronger capacity to buffer and sustain unbalanced D genome chromosomes by the BBAA subgenomes would be in favor of these properties and facilitate the persistence of pentaploid-derived lineages for long enough to ensure successful reversion to euploid hexaploid wheat with incorporated genetic variations from the tetraploid wheats. Analogically, a stronger inclusiveness to whole chromosome aneuploidies for protracted period may generate additional genomic variations as documented in yeast (Sheltzer et al., 2011). Together, repeated hybridizations with diverse accessions of its tetraploid emmer wheat progenitor would apparently contribute to the higher than expected level of genetic diversities seen in hexaploid common wheat. These introgressed or *de novo* generated genetic diversities (due to protracted states of aneuploidy) might have served as raw materials for evolving the remarkable adaptability of hexaploid common wheat to a wide spectrum of climatic and environmental conditions around the world.

By using the pentaploids as intermediates, several agriculturally important traits have been reciprocally introgressed between tetraploid *durum* wheat and hexaploid common wheat (Xu et al., 2013; Kalous et al., 2015; Han et al., 2016). However, it is recognized that the efficacy of these endeavors is cross (genotype) dependent. Further understanding of the genetic basis underlying the trait of capacitating imbalanced D chromosomes by the BBAA component will undoubtedly enable more judicious designing of the crosses and increase the breeding efficiency.

## Author Contributions

BL and XD conceived and designed the project. XD and YS performed the experiments. FW and BL contributed reagents, materials, and analysis tools. XD, FW, and BL wrote the paper. ZL, YW, and AZ contributed with essential suggestions and data analyses in the course of the project. All authors contributed to manuscript revision, proofreading, and approval of the final manuscript.

## Conflict of Interest Statement

The authors declare that the research was conducted in the absence of any commercial or financial relationships that could be construed as a potential conflict of interest.

## Footnotes

1 https://CRAN.R-project.org/package=pheatmap

## References

[B1] Birchler J. A. Bhadra U. Bhadra M. P. Auger D. L. (2001). Dosage-dependent gene regulation in multicellular eukaryotes: implications for dosage compensation, aneuploid syndromes, and quantitative traits. *Dev. Biol.* 234 275–288. 10.1006/dbio.2001.0262 11396999

[B2] Chester M. Gallagher J. P. Symonds V. V. da Silva A. V. C. Mavrodiev E. V. Leitch A. R. (2012). Extensive chromosomal variation in a recently formed natural allopolyploid species, *Tragopogon miscellus* (Asteraceae). *Proc. Natl. Acad. Sci. U.S.A.* 109 1176–1181. 10.1073/pnas.1112041109 22228301 PMC3268322

[B3] Dubcovsky J. Dvorak J. (2007). Genome plasticity a key factor in the success of polyploid wheat under domestication. *Science* 316 1862–1866. 10.1126/science.1143986 17600208 PMC4737438

[B4] Eberhard F. S. Zhang P. Lehmensiek A. Hare R. A. Simpfendorfer S. Sutherland M. W. (2010). Chromosome composition of an F2 *Triticum aestivum*× *T. turgidum* spp. durum cross analysed by DArT markers and MCFISH. *Crop Pasture Sci.* 61 619–624. 10.1071/CP10131 20583599

[B5] Feldman M. Levy A. A. (2012). Genome evolution due to allopolyploidization in wheat. *Genetics* 192 763–774. 10.1534/genetics.112.146316 23135324 PMC3522158

[B6] Feldman M. Lupton F. G. H. Miller T. E. (1995). “Evolution of crop plants,” in *Wheats* eds Smartt J. Simmonds N. W. (London: Longman Scientific & Technical) 184–192.

[B7] Gao L. Diarso M. Zhang A. Zhang H. Dong Y. Liu L. (2016). Heritable alteration of DNA methylation induced by whole-chromosome aneuploidy in wheat. *New Phytol.* 209 364–375. 10.1111/nph.13595 26295562

[B8] Gasch A. P. Hose J. Newton M. A. Sardi M. Yong M. Wang Z. (2016). Further support for aneuploidy tolerance in wild yeast and effects of dosage compensation on gene copy-number evolution. *eLife* 5:e14409. 10.7554/eLife.14409 26949252 PMC4798956

[B9] Han C. Zhang P. Ryan P. R. Rathjen T. M. Yan Z. Delhaize E. (2016). Introgression of genes from bread wheat enhances the aluminium tolerance of durum wheat. *Theor. Appl. Genet.* 129 729–739. 10.1007/s00122-015-2661-3 26747046

[B10] Henry I. M. Dilkes B. P. Comai L. (2007). Genetic basis for dosage sensitivity in *Arabidopsis thaliana*. *PLoS Genet.* 3:e70. 10.1371/journal.pgen.0030070 17465685 PMC1857734

[B11] Henry I. M. Dilkes B. P. Miller E. S. Burkart-Waco D. Comai L. (2010). Phenotypic consequences of aneuploidy in *Arabidopsis thaliana*. *Genetics* 186 1231–1245. 10.1534/genetics.110.121079 20876566 PMC2998307

[B12] Henry I. M. Dilkes B. P. Young K. Watson B. Wu H. Comai L. (2005). Aneuploidy and genetic variation in the *Arabidopsis thaliana* triploid response. *Genetics* 170 1979–1988. 10.1534/genetics.104.037788 15944363 PMC1449780

[B13] Hintze J. L. Nelson R. D. (1998). Violin plots: a box plot-density trace synergism. *Am. Stat.* 52 181–184.

[B14] Hose J. Yong C. M. Sardi M. Wang Z. Newton M. A. Gasch A. P. (2015). Dosage compensation can buffer copy-number variation in wild yeast. *eLife* 4:e05462 10.7554/eLife.05462.001 PMC4448642 25955966

[B15] Huang S. Sirikhachornkit A. Su X. Faris J. Gill B. Haselkorn R. (2002). Genes encoding plastid acetyl-CoA carboxylase and 3-phosphoglycerate kinase of the *Triticum/Aegilops* complex and the evolutionary history of polyploid wheat. *Proc. Natl. Acad. Sci. U.S.A.* 99 8133–8138. 10.1073/pnas.072223799 12060759 PMC123033

[B16] Kalous J. R. Martin J. M. Sherman J. D. Heo H.-Y. Blake N. K. Lanning S. P. (2015). Impact of the D genome and quantitative trait loci on quantitative traits in a spring durum by spring bread wheat cross. *Theor. Appl. Genet.* 128 1799–1811. 10.1007/s00122-015-2548-3 26037088

[B17] Kashkush K. Feldman M. Levy A. A. (2002). Gene loss, silencing and activation in a newly synthesized wheat allotetraploid. *Genetics* 160 1651–1659. 11973318 10.1093/genetics/160.4.1651 PMC1462064

[B18] Kashkush K. Feldman M. Levy A. A. (2003). Transcriptional activation of retrotransposons alters the expression of adjacent genes in wheat. *Nat. Genet.* 33 102–106. 10.1038/ng1063 12483211

[B19] Kaya A. Gerashchenko M. V. Seim I. Labarre J. Toledano M. B. Gladyshev V. N. (2015). Adaptive aneuploidy protects against thiol peroxidase deficiency by increasing respiration via key mitochondrial proteins. *Proc. Natl. Acad. Sci. U.S.A.* 112 10685–10690. 10.1073/pnas.1505315112 26261310 PMC4553801

[B20] Kerber E. R. (1964). Wheat: reconstitution of the tetraploid component (AABB) of hexaploids. *Science* 143 253–255. 10.1126/science.143.3603.253 17753152

[B21] Kihara H. (1925). Weitere über die pentaploiden *Triticum* - Bastarde. I. *Jpn. J. Bot.* 2 299–305.

[B22] Kihara H. (1944). Discovery of the DD-analyser, one of the ancestors of *Triticum vulgare*. *Agric. Hortic.* 19 13–14. 10.1016/j.jgg.2011.07.002 21867963

[B23] Letourneau A. Santoni F. A. Bonilla X. Sailani M. R. Gonzalez D. Kind J. (2014). Domains of genome-wide gene expression dysregulation in down’s syndrome. *Nature* 508 345–350. 10.1038/nature13200 24740065

[B24] Li A. L. Geng S. F. Zhang L. Q. Liu D. C. Mao L. (2015). Making the bread: insights from newly synthesized allohexaploid wheat. *Mol. Plant* 8 847–859. 10.1016/j.molp.2015.02.016 25747845

[B25] Liu C. Yang X. Zhang H. Wang X. Zhang Z. Bian Y. (2015). Genetic and epigenetic modifications to the BBAA component of common wheat during its evolutionary history at the hexaploid level. *Plant Mol. Biol.* 88 53–64. 10.1007/s11103-015-0307-0 25809554

[B26] Martin A. Simpfendorfer S. Hare R. A. Eberhard F. S. Sutherland M. W. (2011). Retention of D genome chromosomes in pentaploid wheat crosses. *Heredity* 107 315–319. 10.1038/hdy.2011.17 21427754 PMC3182498

[B27] Matzke M. A. Florian Mette M. Kanno T. Matzke A. J. M. (2003). Does the intrinsic instability of aneuploid genomes have a causal role in cancer? *Trends Genet.* 19 253–256. 12711216 10.1016/s0168-9525(03)00057-x

[B28] McFadden E. S. Sears E. R. (1946). The origin of *Triticum spelta* and its free-threshing hexaploid relatives. *J. Hered.* 37 81–89. 10.1093/oxfordjournals.jhered.a105590 20985728

[B29] Megarbane A. Ravel A. Mircher C. Sturtz F. Grattau Y. Rethore M.-O. (2009). The 50th anniversary of the discovery of trisomy 21: the past, present, and future of research and treatment of Down syndrome. *Genet. Med.* 11 611–616. 10.1097/GIM.0b013e3181b2e34c 19636252

[B30] Millet C. Ausiannikava D. Le Bihan T. Granneman S. Makovets S. (2015). Cell populations can use aneuploidy to survive telomerase insufficiency. *Nat. Commun.* 6:8664. 10.1038/ncomms9664 26489519 PMC4627575

[B31] Padmanaban S. Sutherland M. W. Knight N. L. Martin A. (2017a). Genome inheritance in populations derived from hexaploid/tetraploid and tetraploid/hexaploid wheat crosses. *Mol. Breed.* 37:48. 10.1007/s11032-017-0647-3 21427754

[B32] Padmanaban S. Zhang P. Hare R. A. Sutherland M. W. Martin A. (2017b). Pentaploid wheat hybrids: applications, characterisation, and challenges. *Front. Plant Sci.* 8:358. 10.3389/fpls.2017.00358 28367153 PMC5355473

[B33] Padmanaban S. Zhang P. Sutherland M. W. Knight N. L. Martin A. (2018). A cytological and molecular analysis of D-genome chromosome retention following F2–F6 generations of hexaploid × tetraploid wheat crosses. *Crop Pasture Sci.* 69 121–130. 10.1071/CP17240

[B34] Pavelka N. Rancati G. Zhu J. Bradford W. D. Saraf A. Florens L. (2010). Aneuploidy confers quantitative proteome changes and phenotypic variation in budding yeast. *Nature* 468 321–325. 10.1038/nature09529 20962780 PMC2978756

[B35] Ramsey J. Schemske D. W. (1998). Pathways, mechanisms, and rates of polyploid formation in flowering plants. *Annu. Rev. Ecol. Syst.* 29 467–501. 10.1104/pp.16.01768 28034953 PMC5291013

[B36] Rancati G. Pavelka N. Fleharty B. Noll A. Allen R. Walton K. (2008). Aneuploidy and polyploidy underlie adaptive evolution of yeast cells deprived of a conserved cytokinesis motor. *Cell* 135 879–893. 10.1016/j.cell.2008.09.039 19041751 PMC2776776

[B37] Rayburn A. L. Gill B. S. (1986). Isolation of a genome-specific repeated DNA sequence from *Aegilops squarrosa*. *Plant Mol. Biol. Rep.* 4 102–109. 10.1007/BF02732107

[B38] Rutledge S. D. Cimini D. (2016). Consequences of aneuploidy in sickness and in health. *Curr. Opin. Cell Biol.* 40 41–46. 10.1016/j.ceb.2016.02.003 26919076

[B39] Salamini F. Ozkan H. Brandolini A. Schafer-Pregl R. Martin W. (2002). Genetics and geography of wild cereal domestication in the near east. *Nat. Rev. Genet.* 3 429–441. 10.1038/nrg817 12042770

[B40] Sansregret L. Swanton C. (2017). The role of aneuploidy in cancer evolution. *Cold Spring Harb. Perspect. Med.* 7:a028373. 10.1101/cshperspect.a028373 28049655 PMC5204330

[B41] Sax K. (1922). Sterility in wheat hybrids. III. Endosperm development and F2 sterility. *Genetics* 7 553–558. 17245992 10.1093/genetics/7.6.553 PMC1200544

[B42] Sears E. R. (1944). Cytogenetic studies with polyploid species of wheat. II. Additional chromosomal aberrations in *Triticum vulgare.* *Genetics* 29 232–246. 17247118 10.1093/genetics/29.3.232 PMC1209244

[B43] Sheltzer J. M. Blank H. M. Pfau S. J. Tange Y. George B. M. Humpton T. J. (2011). Aneuploidy drives genomic instability in yeast. *Science* 333 1026–1030. 10.1126/science.1206412 21852501 PMC3278960

[B44] Soltis P. S. Marchant D. B. Van de Peer Y. Soltis D. E. (2015). Polyploidy and genome evolution in plants. *Curr. Opin. Genet. Dev.* 35 119–125. 10.1016/j.gde.2015.11.003 26656231

[B45] Torres E. M. Springer M. Amon A. (2016). No current evidence for widespread dosage compensation in *S. cerevisiae*. *eLife* 5:e10996. 10.7554/eLife.10996 26949255 PMC4798953

[B46] Torres E. M. Williams B. R. Amon A. (2008). Aneuploidy: cells losing their balance. *Genetics* 179 737–746. 10.1534/genetics.108.090878 18558649 PMC2429870

[B47] Wang H. Y. Liu D. C. Yan Z. H. Wei Y. M. Zheng Y. L. (2005). Cytological characteristics of F2 hybrids between *Triticum aestivum* L. and *T. durum* Desf. with reference to wheat breeding. *J. Appl. Genet.* 46 365–369. 16278508

[B48] Wu Y. Sun Y. Sun S. Li G. Wang J. Wang B. (2018). Aneuploidization under segmental allotetraploidy in rice and its phenotypic manifestation. *Theor. Appl. Genet.* 131 1273–1285. 10.1007/s00122-018-3077-7 29478186 PMC5945760

[B49] Xiong Z. Gaeta R. T. Pires J. C. (2011). Homoeologous shuffling and chromosome compensation maintain genome balance in resynthesized allopolyploid *Brassica napus*. *Proc. Natl. Acad. Sci. U.S.A.* 108 7908–7913. 10.1073/pnas.1014138108 21512129 PMC3093481

[B50] Xu L. S. Wang M. N. Cheng P. Kang Z. S. Hulbert S. H. Chen X. M. (2013). Molecular mapping of Yr53, a new gene for stripe rust resistance in durum wheat accession PI 480148 and its transfer to common wheat. *Theor. Appl. Genet.* 126 523–533. 10.1007/s00122-012-1998-0 23090143

[B51] Zhang A. Li N. Gong L. Gou X. Wang B. Deng X. (2017). Global analysis of gene expression in response to whole-chromosome aneuploidy in hexaploid wheat. *Plant Physiol.* 175 828–847. 10.1104/pp.17.00819 28821592 PMC5619904

[B52] Zhang H. Bian Y. Gou X. Zhu B. Xu C. Qi B. (2013). Persistent whole-chromosome aneuploidy is generally associated with nascent allohexaploid wheat. *Proc. Natl. Acad. Sci. U.S.A.* 110 3447–3452. 10.1073/pnas.1300153110 23401544 PMC3587266

[B53] Zhang H. Gou X. Zhang A. Wang X. Zhao N. Dong Y. (2016). Transcriptome shock invokes disruption of parental expression-conserved genes in tetraploid wheat. *Sci. Rep.* 6:26363. 10.1038/srep26363 27198893 PMC4873831

[B54] Zhang H. Zhu B. Qi B. Gou X. Dong Y. Xu C. (2014). Evolution of the BBAA component of bread wheat during its history at the allohexaploid level. *Plant Cell* 26 2761–2776. 10.1105/tpc.114.128439 24989045 PMC4145112

[B55] Zhao N. Xu L. Zhu B. Li M. Zhang H. Qi B. (2011). Chromosomal and genome-wide molecular changes associated with initial stages of allohexaploidization in wheat can be transit and incidental. *Genome* 54 692–699. 10.1139/g11-028 21797821

