# Supplementary material for: The Capacity to Buffer and Sustain Imbalanced D-Subgenome Chromosomes by the BBAA Component of Hexaploid Wheat Is an Evolved Dominant Trait
Source: Front Plant Sci. 2018 Aug 7;9:1149. doi: 10.3389/fpls.2018.01149 (PMC6090280; doi:10.3389/fpls.2018.01149)
Supplement: Supplementary file 1 [file Table_1.DOCX]

**Table S1**. Summary of cohorts showing numerical chromosome variations (NCVs) in the B and/or A subgenome chromosomes, which are or are not accompanied with NCVs of the D chromosomes

| Involved Subgenome | XX329 × ETW | TAA10 × ETW | TAA10 × TTR13 | SHW × TTR13 |
| --- | --- | --- | --- | --- |
| A-Subgenome | -5A, -6A, +1A | -1A, +1A (2) | -1A (4), -6A, +1A (3), +2A | -4A, -5A, +2A |
| B-Subgenome | -6B (2), -7B (2), +1B, +5B | -4B | -5B, +1B, +3B | +5B, +6B |
| D-Subgenome | +5D (3) | +4D | +1D | -- |
| Inter-subgenome | +1A & +6A & +6B, -4B & +5D, -3A & -3B | -- | +1A &+6D | -- |

Note: Symbols – and + refer to loss and gain of the pertinent chromosomes, respectively.

Numericals in the parenthesis represent the numbers of cohorts showing the NCVs greater than one.
