# Supplementary material for: The Capacity to Buffer and Sustain Imbalanced D-Subgenome Chromosomes by the BBAA Component of Hexaploid Wheat Is an Evolved Dominant Trait
Source: Front Plant Sci. 2018 Aug 7;9:1149. doi: 10.3389/fpls.2018.01149 (PMC6090280; doi:10.3389/fpls.2018.01149)
Supplement: Supplementary file 2 [file Table_2.DOCX]

**Table S2.** Types of structural chromosome variations (SCVs) and the numbers of cohorts manifesting the specific SCVs

| Type of SCV | Involved Subgenome | XX329 × ETW | TAA10 × ETW | TAA10 × TTR13 | SHW × TTR13 |
| --- | --- | --- | --- | --- | --- |
| Telocentric | A | 4AS | 1AS | -- | -- |
|  | B | -- | -- | -- | 5BS |
|  | D | 1DS (2), 2DL, 3DS, 3DL, 4DS, 5DS, 5DL, 6DS, 7DS (3), 7DL (4) | 1DL, 2DL (2), 3DS, 3DL (2), 5DS (2), 5DL, 6DS, 7DS (2), 3DL & 7DL | 2DS (3), 3DS (2), 3DL (2), 5DS (2), 5DL, 6DS (2), 6DL (2), 7DL (5) | 1DS (9), 1DL (5), 2DS (2), 2DL, 3DS (2), 4DS, 5DL, 7DS, 7DL (2) |
| Translocation | Inter-subgenome | 2A-D, 6A-D, 7A-3DL, 2D-A, 3D-A, 6B-7DS | 6A-D, 1D-A (2), 5D-B | 2D-A (3), 2D-A & 2DS.3DS | 7DS.6BL |
|  | Intra-subgenome | 2DS.1DL, 2DS.3DS, 4DL.7DL, 1D and 3D reciprocal translocation, 1D and 7D reciprocal translocation | 4DS.2DL, 6DS.5DL, 1A and 1D reciprocal translocation | 2DS.1DL, 6DS.7DL, 2D and 6D reciprocal translocation | 2DS.3DS, 2DS.7DS, 2DS.7DL (2), 3DS.2DL,1DS.4DL |
| Isochromosomes | D | 2DS.2DS, 3DS.3DS | 2DL.2DL (2) | 3DL.3DL (3), 5DL.5DL (2), 6DS.6DS | 3DS.3DS (2), 5DL.5DL, 7DS.7DS |
| Telocentric and Translocation | Inter/Intra-subgenome | 3DS & 1DL.3DL | 6DS & 1D-A, 6DL & 5DL.7DL | 6DL& 6DS.5DL | -- |
| Translocation and NCV of A or B chromosome | Inter/Intra-subgenome | 2A-D & +2A, 5A-D & +5A | -- | A-D & -1A | -- |
| Telocentric and NCV of A or B chromosome | Inter/Intra-subgenome | -- | -- | 5DS & -2B | -- |
| Isochromosome and NCV of A or B chromosome | Inter/Intra-subgenome | -- | -- | 6DS.6DS & -1A | -- |

Note: Symbols – and + represents loss of a chromosome (monosomic) and gain of a chromosome (trisomic), respectively.

Numericals in the brackets refer to the numbers of plants with the specific SCVs, which are greater than one.
